# Supplementary material for: Eye morphogenesis driven by epithelial flow into the optic cup facilitated by modulation of bone morphogenetic protein
Source: eLife. 2015 Feb 24;4:e05216. doi: 10.7554/eLife.05216 (PMC4337729; doi:10.7554/eLife.05216)
Supplement: Source code 1. — Source Code .zip contains: Plugin: imageJ plugin for visualization of dorso-ventral movements (please see ‘Materials and methods’ section: quantification of dorso-ventral movement). Macro: imageJ macro for counting of labeled pixels (please see ‘Materials and methods’ section: quantification of dorso-ventral movement). DOI: http://dx.doi.org/10.7554/eLife.05216.024 [file elife05216s001.zip › 16-10-2014-SR-eLife-05216R1_Supplemental_file_macro.docx]

**Supplemental file macro:**

//Color counter

setBatchMode(true);

zn = 113;

yn = 704;

xn= 544;

colorCounterBlue = 0;

colorCounterCyan = 0;

colorCounterGreen = 0;

colorCounterLightgreen = 0;

colorCounterYellow = 0;

colorCounterOrange = 0;

colorCounterRed = 0;

colorCounterPink = 0;

colorCounterWhite = 0;

for (z=1; z<=zn; z++){

for (y=1; y<=yn; y++){

for (x=1; x<=xn; x++){

setSlice(z);

value = getPixel(x,y);

if (value>=32 && value<=47) colorCounterBlue ++;

else if (value>=80 && value<=95) colorCounterCyan++;

else if (value>=96 && value<=111) colorCounterGreen++;

else if (value>=112 && value<=127) colorCounterLightgreen++;

else if (value>=128 && value<=143) colorCounterYellow++;

else if (value>=160 && value<=175) colorCounterOrange++;

else if (value>=192 && value<=207) colorCounterRed++;

else if (value>=208 && value<=223) colorCounterPink++;

else if (value>=240 && value<=255) colorCounterWhite++;

}

}

}

Zcolors = colorCounterCyan + colorCounterBlue + colorCounterLightgreen + colorCounterYellow + colorCounterOrange + colorCounterPink;

Non_Zcolors = colorCounterRed + colorCounterGreen;

total = colorCounterCyan + colorCounterBlue + colorCounterLightgreen + colorCounterYellow + colorCounterOrange + colorCounterPink + colorCounterRed + colorCounterGreen + colorCounterWhite;

Z_share = Zcolors/total;

print("Color: Number of pixels:");

print("Blue: "+colorCounterBlue);

print("Cyan: "+colorCounterCyan);

print("Green: "+colorCounterGreen);

print("Lightgreen: "+colorCounterLightgreen);

print("Yellow: "+colorCounterYellow);

print("Orange: "+colorCounterOrange);

print("Red: "+colorCounterRed);

print("Pink: "+colorCounterPink);

print("White: "+colorCounterWhite);

print("Zcolors: "+Zcolors);

print("Non_Zcolors: "+Non_Zcolors);

print("Total number of colored pixels: "+total);

print("Z_share: "+Z_share);
