# Supplementary material for: Eye morphogenesis driven by epithelial flow into the optic cup facilitated by modulation of bone morphogenetic protein
Source: eLife. 2015 Feb 24;4:e05216. doi: 10.7554/eLife.05216 (PMC4337729; doi:10.7554/eLife.05216)
Supplement: Source code 1. — Source Code .zip contains: Plugin: imageJ plugin for visualization of dorso-ventral movements (please see ‘Materials and methods’ section: quantification of dorso-ventral movement). Macro: imageJ macro for counting of labeled pixels (please see ‘Materials and methods’ section: quantification of dorso-ventral movement). DOI: http://dx.doi.org/10.7554/eLife.05216.024 [file elife05216s001.zip › 16-10-2014-SR-eLife-05216R1_Supplemental_file_plugin.docx]

**Supplemental file plugin:**

import ij.*;

import ij.process.*;

import ij.gui.*;

//import java.awt.*;

import ij.plugin.*;

import ij.plugin.frame.*;

import ij.ImagePlus.*;

import java.lang.*;

import java.util.*;

import java.io.*;

import ij.io.*;

import ij.gui.GenericDialog.*;

public class Eye_Conditional_Visualization3 implements PlugIn {

public String color_t1;

public String color_t0;

public void run(String arg) {

int nx, ny, nz, k, sFrame, eFrame;

boolean Frame1, smooth, seg, fForm, flatMap, ventralSide, dorsalSide, elongated;

boolean alreadyInside=false;

boolean furrow=false;

String inputPath, outputPath, zone;

double psiEntryThreshold, psiMainThreshold, thetaEntryThreshold, thetaMainThreshold, psiSwitchThreshold, thetaSwitchThreshold;

double xCenter, yCenter, zCenter, xCenterS, yCenterS, zCenterS, quotient, xM, yM, xP1circ, yP1circ, xP2circ, yP2circ, xP3circ, yP3circ;

double circleRadius, radiusThreshold, thetaP1, thetaP2, thetaP3, topP1, topP2, topP3, radDiff;

double xP1, yP1, xP2, yP2, xP3, yP3;

int x_flat, y_flat;

int redcounter, whitecounter, greencounter, lightgreencounter, yellowcounter, bluecounter, cyancounter, orangecounter, pinkcounter;

GenericDialog inputParams = new GenericDialog("Conditional 4D Cell Flow Visualization");

inputParams.addStringField("Enter text file directory: ", "/media/lucas_schuetz/IMANALYSIS1/Eye_Development/Output_Flow_turned/",40);

inputParams.addStringField("Enter output directory: ", "/media/lucas_schuetz/IMANALYSIS1/Eye_Development/Conditional_Visualization_turned/Run_1/", 40);

inputParams.addMessage("---------------------Dimensions------------------------------------");

inputParams.addNumericField("X-width: ", 544, 10);

inputParams.addNumericField("Y-width: ", 704, 10);

inputParams.addNumericField("Z-slices: ", 44, 10);

inputParams.addNumericField("X-center of embryo: ", 399, 10);

inputParams.addNumericField("Y-center of embryo: ", 321, 10);

inputParams.addNumericField("Z-center of embryo: ", 22, 10);

inputParams.addNumericField("Starting Frame: ", 41, 10);

inputParams.addNumericField("Last Frame: ", 44, 10);

inputParams.addCheckbox("First Frame", true);

inputParams.addMessage("---------------------Visualization parameters----------------------");

inputParams.addNumericField("Delta psi entry Threshold: ", 0.005, 10);

inputParams.addNumericField("Delta psi main Threshold: ", 0.002, 10);

inputParams.addNumericField("Delta theta entry Threshold: ", 0.005, 10);

inputParams.addNumericField("Delta theta main Threshold: ", 0.002, 10);

inputParams.addNumericField("Delta psi switch color Threshold: ", 0.005, 10);

inputParams.addNumericField("Delta theta switch color Threshold: ", 0.005, 10);

inputParams.addCheckbox("Enable smoothing", false);

inputParams.addCheckbox("Enable object segmentation", false);

inputParams.addCheckbox("Enable furrow labeling", false);

inputParams.addCheckbox("Create flat map (needs furrow labeling)", false);

inputParams.addCheckbox("Cut at ventral side (needs flat map)", false);

inputParams.addCheckbox("Cut at dorsal side (needs flat map)", false);

inputParams.addCheckbox("Show dorsal side as elongation", false);

inputParams.showDialog();

if (inputParams.wasCanceled()){

return;

}

inputPath= inputParams.getNextString();

outputPath= inputParams.getNextString();

nx= (int) inputParams.getNextNumber();

ny= (int) inputParams.getNextNumber();

nz= (int) inputParams.getNextNumber();

xCenterS= inputParams.getNextNumber();

yCenterS= inputParams.getNextNumber();

zCenterS= inputParams.getNextNumber();

sFrame= (int) inputParams.getNextNumber();

eFrame= (int) inputParams.getNextNumber();

Frame1= inputParams.getNextBoolean();

psiEntryThreshold= inputParams.getNextNumber();

psiMainThreshold= inputParams.getNextNumber();

thetaEntryThreshold= inputParams.getNextNumber();

thetaMainThreshold= inputParams.getNextNumber();

psiSwitchThreshold= inputParams.getNextNumber();

thetaSwitchThreshold= inputParams.getNextNumber();

smooth= inputParams.getNextBoolean();

seg= inputParams.getNextBoolean();

fForm= inputParams.getNextBoolean();

flatMap = inputParams.getNextBoolean();

ventralSide= inputParams.getNextBoolean();

dorsalSide= inputParams.getNextBoolean();

elongated= inputParams.getNextBoolean();

xP1 = 168;

yP1 = 0;

xP2 = 0;

yP2 = 273;

xP3 = 336;

yP3 = 273;

new File(outputPath+"3D_Embryo/").mkdirs();

new File(outputPath+"Ventral_split/").mkdirs();

new File(outputPath+"Dorsal_split/").mkdirs();

new File(outputPath+"Elongated/").mkdirs();

//int j=1;

for (int j=sFrame; j<=eFrame; j++){

k=j-1;

ImagePlus Framet1_imp = IJ.createImage("Frame_"+j, "RGB black", nx, ny, nz);

IJ.run(Framet1_imp, "8-bit Color", "number=256");

//IJ.run("Threshold...");

IJ.setThreshold(Framet1_imp, 0, 255);

//IJ.run("Brightness/Contrast...");

IJ.run(Framet1_imp, "Apply LUT", "stack");

IJ.run(Framet1_imp, "16_colors_ramp", "");

/*ImagePlus flatFrameV_imp = IJ.createImage("flatFrameV_"+j, "RGB black", 1000, 200, nz);

IJ.run(flatFrameV_imp, "8-bit Color", "number=256");

//IJ.run("Threshold...");

IJ.setThreshold(flatFrameV_imp, 0, 255);

//IJ.run("Brightness/Contrast...");

IJ.run(flatFrameV_imp, "Apply LUT", "stack");

IJ.run(flatFrameV_imp, "16_colors_ramp", "");

ImagePlus flatFrameD_imp = IJ.createImage("flatFrameD_"+j, "RGB black", 1000, 200, nz);

IJ.run(flatFrameD_imp, "8-bit Color", "number=256");

//IJ.run("Threshold...");

IJ.setThreshold(flatFrameD_imp, 0, 255);

//IJ.run("Brightness/Contrast...");

IJ.run(flatFrameD_imp, "Apply LUT", "stack");

IJ.run(flatFrameD_imp, "16_colors_ramp", "");

ImagePlus flatFrameEl_imp = IJ.createImage("flatFrameEl_"+j, "RGB black", 1000, 200, 2*nz);

IJ.run(flatFrameEl_imp, "8-bit Color", "number=256");

//IJ.run("Threshold...");

IJ.setThreshold(flatFrameEl_imp, 0, 255);

//IJ.run("Brightness/Contrast...");

IJ.run(flatFrameEl_imp, "Apply LUT", "stack");

IJ.run(flatFrameEl_imp, "16_colors_ramp", ""); */

ImageStack Framet1_is= Framet1_imp.getStack();

/*ImageStack flatFrameV_is= flatFrameV_imp.getStack();

ImageStack flatFrameD_is= flatFrameD_imp.getStack();

ImageStack flatFrameEl_is= flatFrameEl_imp.getStack(); */

//int[] pixels_t1=(int[])Framet1_is.getPixels();

String path = (outputPath+"3D_Embryo/Frame_"+k+".tif");

ImagePlus imp = (Frame1 == false) ? IJ.openImage(path) : null;

ImageStack Framet0_is = null;

try {

Framet0_is = imp.getImageStack();

} catch (NullPointerException ex) {

IJ.log("Couldn't open file at " + path);

}

//ImagePlus Framet0_imp=IJ.openImage(path);

//ImageStack Framet0_is=Framet0_imp.getStack();

//int pixels_t0[]=(int[])Framet0_is.getPixels();

//add 8bitC procedure if necessary

File file = new File(inputPath+"Timepoint_t0_"+j+"/opticalFlow.txt");

Scanner filestring = null;

try {

filestring= new Scanner(file);

} catch (Exception ex) {

}

List<String> lines = new ArrayList<String>();

int xt1;

int yt1;

int zt1;

int val_t1;

xP1circ = 0;

yP1circ = 0;

xP2circ = 0;

yP2circ = 0;

xP3circ = 0;

yP3circ = 0;

radiusThreshold = 0;

xM=0;

yM=0;

xCenter=xCenterS;

yCenter=yCenterS;

zCenter=zCenterS;

topP1 = nx;

topP2 = nx;

topP3 = nx;

circleRadius=1;

whitecounter = 0;

redcounter = 0;

pinkcounter = 0;

greencounter = 0;

lightgreencounter = 0;

orangecounter = 0;

yellowcounter = 0;

bluecounter = 0;

cyancounter = 0;

int i=0;

for (int z=1; z<=nz; z++){

/* quotient = 2*(xP1circ*(yP2circ-yP3circ)+xP2circ*(yP3circ-yP1circ)+xP3circ*(yP1circ-yP2circ));

xM = ((Math.pow(xP1circ,2)+Math.pow(yP1circ,2))*(yP2circ-yP3circ)+(Math.pow(xP2circ,2)+Math.pow(yP2circ,2))*(yP3circ-yP1circ)+(Math.pow(xP3circ,2)+Math.pow(yP3circ,2))*(yP1circ-yP2circ))/quotient;

yM = ((Math.pow(xP1circ,2)+Math.pow(yP1circ,2))*(xP3circ-xP2circ)+(Math.pow(xP2circ,2)+Math.pow(yP2circ,2))*(xP1circ-xP3circ)+(Math.pow(xP3circ,2)+Math.pow(yP3circ,2))*(xP2circ-xP1circ))/quotient;

circleRadius = Math.sqrt(Math.pow((xM-xP1circ),2)+Math.pow((yM-yP1circ),2));

radiusThreshold = circleRadius-20;

topP1 = nx;

topP2 = nx;

topP3 = nx;

*/

for (int y=1; y<=ny; y++){

for (int x=1; x<=nx; x++){

String rows =filestring.nextLine();

String[] columns = rows.split(" ");

double dx=Double.parseDouble(columns[0]);

double dy=Double.parseDouble(columns[1]);

double dz=Double.parseDouble(columns[2]);

if (dx!=0 || dy!=0 || dz!=0){

xt1 = x + (int) Math.round(dx);

yt1 = y + (int) Math.round(dy);

zt1 = z + (int) Math.round(dz);

//xCenter=xM;

//yCenter=yM;

double u=x-xCenter;

double v=y-yCenter;

double w=z-zCenter;

double dist_t0=Math.sqrt(Math.pow(v,2)+Math.pow(u,2)+Math.pow(w,2));

double dist_t1=Math.sqrt(Math.pow((v+dy),2)+Math.pow((u+dx),2)+Math.pow((w+dz),2));

double ddist=dist_t1-dist_t0;

double psi_t0=Math.atan2(v,u);

double psi_t1=Math.atan2((v+dy),(u+dx));

double dpsi=Math.abs(psi_t1)-Math.abs(psi_t0);

double theta_t0=Math.acos(w/dist_t0);

double theta_t1=Math.acos((w+dz)/dist_t1);

double dtheta=Math.abs(theta_t1)-Math.abs(theta_t0);

//Visualization start

val_t1 = 0;

String color_t1 = null;

String color_t0 = null;

if (Frame1==false){

//Framet0_is.setSlice(z);

int val_t0 = (int) Framet0_is.getVoxel(x,y,z);

if (val_t0>=32 && val_t0<=47) color_t0="blue";

else if (val_t0>=80 && val_t0<=95) color_t0="cyan";

else if (val_t0>=96 && val_t0<=111) color_t0="green";

else if (val_t0>=112 && val_t0<=127) color_t0="lightgreen";

else if (val_t0>=128 && val_t0<=143) color_t0="yellow";

else if (val_t0>=160 && val_t0<=175) color_t0="orange";

else if (val_t0>=192 && val_t0<=207) color_t0="red";

else if (val_t0>=208 && val_t0<=223) color_t0="pink";

else if (val_t0>=240 && val_t0<=255) color_t0="white";

else color_t0="black";

boolean chigh1=false;

boolean chigh2=false;

boolean penalty=false;

boolean assignment=false;

if (val_t0==34 || val_t0==82 || val_t0==98 || val_t0==114 || val_t0==130 || val_t0==162 || val_t0==194 || val_t0==210 || val_t0==242) chigh1=true;

if (val_t0==35 || val_t0==83 || val_t0==99 || val_t0==115 || val_t0==131 || val_t0==163 || val_t0==195 || val_t0==211 || val_t0==243) chigh1=true;

if (val_t0==36 || val_t0==84 || val_t0==100 || val_t0==116 || val_t0==132 || val_t0==164 || val_t0==196 || val_t0==212 || val_t0==244) chigh2=true;

if (val_t0==47 || val_t0==95 || val_t0==111 || val_t0==127 || val_t0==143 || val_t0==175 || val_t0==207 || val_t0==223 || val_t0==255) penalty=true;

//check and assign preliminary color tag for t1

if (dpsi>psiMainThreshold && dtheta<=thetaMainThreshold && dtheta>=-thetaMainThreshold) color_t1="red";

else if (dpsi>psiMainThreshold && dtheta>thetaMainThreshold) color_t1="orange";

else if (dpsi>psiMainThreshold && dtheta<-thetaMainThreshold) color_t1="pink";

else if (dpsi<-psiMainThreshold && dtheta<=thetaMainThreshold && dtheta>=-thetaMainThreshold) color_t1="green";

else if (dpsi<-psiMainThreshold && dtheta>thetaMainThreshold) color_t1="lightgreen";

else if (dpsi<-psiMainThreshold && dtheta<-thetaMainThreshold) color_t1="cyan";

else if (dtheta>thetaMainThreshold && dpsi<=psiMainThreshold && dpsi>=-psiMainThreshold) color_t1="yellow";

else if (dtheta<-thetaMainThreshold && dpsi<=psiMainThreshold && dpsi>=-psiMainThreshold) color_t1="blue";

else color_t1="white";// (dtheta>=-thetaMainThreshold && dtheta <=thetaMainThreshold && dpsi>=-psiMainThreshold && dpsi<=psiMainThreshold)

if (color_t1.equals("blue")) {

if (color_t0.equals("white") || color_t0.equals("black")){ //CASE I: color_t0.equals(white

if (dtheta<-thetaEntryThreshold && dpsi<=psiMainThreshold && dpsi>=-psiMainThreshold) {

val_t1=32; //request high initiation threshold for starting 32=blue starting value

color_t1="blue";

assignment=true;

} else

{

if (val_t0<10) val_t1=240;

else if (val_t0<244) val_t1=val_t0+1; //threshold not reached=> assign white using counter

else val_t1=val_t0;

color_t1="white";

assignment=true;

}

}

else if (val_t0>47 && val_t0<240) //CASE II: different color but white

{

if (color_t0.equals("pink") || color_t0.equals("cyan")) //CASE IIa neighbouring color

{

if (Math.abs(dtheta)>thetaSwitchThreshold)

{

val_t1=32; //request high initiation Threshold for switching 32=blue starting value

color_t1="blue";

assignment=true;

} else

{

if (val_t0!=95 && val_t0!=223) //check for penalty value, if not true assign previous color, else white

{

val_t1=val_t0;

color_t1=color_t0;

assignment=true;

} else

{

val_t1=241;

color_t1="white";

assignment=true;

}

}

} else

{

if (color_t0.equals("red") || color_t0.equals("orange") || color_t0.equals("green") || color_t0.equals("lightgreen") || color_t0.equals("yellow")) //other than neighbouring colors

{

if (chigh2==true) //if high counter: long movement in one direction keep t0 color, assign penalty

{

val_t1=val_t0+11;

color_t1=color_t0;

assignment=true;

} else //if counter low set white

{

val_t1=241;

color_t1="white";

assignment=true;

}

}

}

}

else if (color_t1.equals(color_t0)) //CASE III: same color for t1 and t0

{

if (val_t0<36) val_t1=val_t0+1; //counter limit

else if (val_t0==47) val_t1=32; //important for penalty reset

else val_t1=36;

color_t1="blue";

assignment=true;

}

}

else if (color_t1.equals("cyan"))

{

if (color_t0.equals("white") || color_t0.equals("black")) //CASE I: color_t0.equals(white

{

if (dpsi<-psiEntryThreshold || dtheta<-thetaEntryThreshold)

{

val_t1=80; //request high initiation Threshold for starting 80=cyan starting value

color_t1="cyan";

assignment=true;

} else

{

if (val_t0<10) val_t1=240;

else if (val_t0<244) val_t1=val_t0+1; //Threshold not reached=> assign white using counter

else val_t1=val_t0;

color_t1="white";

assignment=true;

}

}

else if (val_t0<80 || (val_t0>95 && val_t0<240)) //CASE II: different color but white

{

if (color_t0.equals("blue") || color_t0.equals("green")) //CASE IIa neighbouring color

{

if (Math.abs(dtheta)>thetaSwitchThreshold && Math.abs(dpsi)>psiSwitchThreshold)

{

val_t1=80; //request high initiation Threshold for switching 80=cyan starting value

color_t1="cyan";

assignment=true;

} else

{

if (val_t0!=47 && val_t0!=111) //check for penalty value, if not true assign previous color, else white

{

val_t1=val_t0;

color_t1=color_t0;

assignment=true;

} else

{

val_t1=241;

color_t1="white";

assignment=true;

}

}

} else

{

if (color_t0.equals("red") || color_t0.equals("orange") || color_t0.equals("pink") || color_t0.equals("yellow") || color_t0.equals("lightgreen")) //other than neighbouring colors

{

if (chigh2==true) //if high counter: long movement in one direction keep t0 color, assign penalty

{

val_t1=val_t0+11;

color_t1=color_t0;

assignment=true;

} else //if counter low set white

{

val_t1=241;

color_t1="white";

assignment=true;

}

}

}

}

else if (color_t1.equals(color_t0)) //CASE III: same color for t1 and t0

{

if (val_t0<84) val_t1=val_t0+1; //counter limit

else if (val_t0==95) val_t1=80; //important for penalty reset

else val_t1=84;

color_t1="cyan";

assignment=true;

}

}

else if (color_t1.equals("green"))

{

if (color_t0.equals("white") || color_t0.equals("black")) //CASE I: color_t0.equals(white

{

if (dpsi<-psiEntryThreshold && dtheta<=thetaMainThreshold && dtheta>=-thetaMainThreshold)

{

val_t1=96; //request high initiation Threshold for starting 96=green starting value

color_t1="green";

assignment=true;

} else

{

if (val_t0<10) val_t1=240;

else if (val_t0<244) val_t1=val_t0+1; //Threshold not reached=> assign white using counter

else val_t1=val_t0;

color_t1="white";

assignment=true;

}

}

else if (val_t0<96 || (val_t0>111 && val_t0<240)) //CASE II: different color but white

{

if (color_t0.equals("lightgreen") || color_t0.equals("cyan")) //CASE IIa neighbouring color

{

if (Math.abs(dpsi)>psiSwitchThreshold)

{

val_t1=96; //request high initiation Threshold for switching 96=green starting value

color_t1="green";

assignment=true;

} else

{

if (val_t0!=95 && val_t0!=127) //check for penalty value, if not true assign previous color, else white

{

val_t1=val_t0;

color_t1=color_t0;

assignment=true;

} else

{

val_t1=241;

color_t1="white";

assignment=true;

}

}

} else

{

if (color_t0.equals("red") || color_t0.equals("orange") || color_t0.equals("blue") || color_t0.equals("pink") || color_t0.equals("yellow")) //other than neighbouring colors

{

if (chigh2==true) //if high counter: long movement in one direction keep t0 color, assign penalty

{

val_t1=val_t0+11;

color_t1=color_t0;

assignment=true;

} else //if counter low set white

{

val_t1=241;

color_t1="white";

assignment=true;

}

}

}

}

else if (color_t1.equals(color_t0)) //CASE III: same color for t1 and t0

{

if (val_t0<100) val_t1=val_t0+1; //counter limit

else if (val_t0==111) val_t1=96; //important for penalty reset

else val_t1=100;

color_t1="green";

assignment=true;

}

}

else if (color_t1.equals("lightgreen"))

{

if (color_t0.equals("white") || color_t0.equals("black")) //CASE I: color_t0.equals(white

{

if (dpsi<-psiEntryThreshold || dtheta>thetaEntryThreshold)

{

val_t1=112; //request high initiation Threshold for starting 112=lightgreen starting value

color_t1="lightgreen";

assignment=true;

} else

{

if (val_t0<10) val_t1=240;

else if (val_t0<244) val_t1=val_t0+1; //Threshold not reached=> assign white using counter

else val_t1=val_t0;

color_t1="white";

assignment=true;

}

}

else if (val_t0<112 || (val_t0>127 && val_t0<240)) //CASE II: different color but white

{

if (color_t0.equals("green") || color_t0.equals("yellow")) //CASE IIa neighbouring color

{

if (Math.abs(dtheta)>thetaSwitchThreshold && Math.abs(dpsi)>psiSwitchThreshold)

{

val_t1=112; //request high initiation Threshold for switching 112=lightgreen starting value

color_t1="lightgreen";

assignment=true;

} else

{

if (val_t0!=111 && val_t0!=143) //check for penalty value, if not true assign previous color, else white

{

val_t1=val_t0;

color_t1=color_t0;

assignment=true;

} else

{

val_t1=241;

color_t1="white";

assignment=true;

}

}

} else

{

if (color_t0.equals("red") || color_t0.equals("orange") || color_t0.equals("blue") || color_t0.equals("pink") || color_t0.equals("cyan")) //other than neighbouring colors

{

if (chigh2==true) //if high counter: long movement in one direction keep t0 color, assign penalty

{

val_t1=val_t0+11;

color_t1=color_t0;

assignment=true;

} else //if counter low set white

{

val_t1=241;

color_t1="white";

assignment=true;

}

}

}

}

else if (color_t1.equals(color_t0)) //CASE III: same color for t1 and t0

{

if (val_t0<116) val_t1=val_t0+1; //counter limit

else if (val_t0==127) val_t1=112; //important for penalty reset

else val_t1=116;

color_t1="lightgreen";

assignment=true;

}

}

else if (color_t1.equals("yellow"))

{

if (color_t0.equals("white") || color_t0.equals("black")) //CASE I: color_t0.equals(white

{

if (dtheta>thetaEntryThreshold && dpsi<=psiMainThreshold && dpsi>=-psiMainThreshold)

{

val_t1=128; //request high initiation Threshold for starting 128=yellow starting value

color_t1="yellow";

assignment=true;

} else

{

if (val_t0<10) val_t1=240;

else if (val_t0<244) val_t1=val_t0+1; //Threshold not reached=> assign white using counter

else val_t1=val_t0;

color_t1="white";

assignment=true;

}

}

else if (val_t0<128 || (val_t0>143 && val_t0<240)) //CASE II: different color but white

{

if (color_t0.equals("lightgreen") || color_t0.equals("orange")) //CASE IIa neighbouring color

{

if (Math.abs(dtheta)>thetaSwitchThreshold)

{

val_t1=128; //request high initiation Threshold for switching 128=yellow starting value

color_t1="yellow";

assignment=true;

} else

{

if (val_t0!=127 && val_t0!=175) //check for penalty value, if not true assign previous color, else white

{

val_t1=val_t0;

color_t1=color_t0;

assignment=true;

} else

{

val_t1=241;

color_t1="white";

assignment=true;

}

}

} else

{

if (color_t0.equals("red") || color_t0.equals("pink") || color_t0.equals("green") || color_t0.equals("blue") || color_t0.equals("cyan")) //other than neighbouring colors

{

if (chigh2==true) //if high counter: long movement in one direction keep t0 color, assign penalty

{

val_t1=val_t0+11;

color_t1=color_t0;

assignment=true;

} else //if counter low set white

{

val_t1=241;

color_t1="white";

assignment=true;

}

}

}

}

else if (color_t1.equals(color_t0)) //CASE III: same color for t1 and t0

{

if (val_t0<132) val_t1=val_t0+1; //counter limit

else if (val_t0==143) val_t1=128; //important for penalty reset

else val_t1=132;

color_t1="yellow";

assignment=true;

}

}

else if (color_t1.equals("orange"))

{

if (color_t0.equals("white") || color_t0.equals("black")) //CASE I: color_t0.equals(white

{

if (dpsi>psiEntryThreshold || dtheta>thetaEntryThreshold)

{

val_t1=160; //request high initiation Threshold for starting 160=orange starting value

color_t1="orange";

assignment=true;

} else

{

if (val_t0<10) val_t1=240;

else if (val_t0<244) val_t1=val_t0+1; //Threshold not reached=> assign white using counter

else val_t1=val_t0;

color_t1="white";

assignment=true;

}

}

else if (val_t0<160 || (val_t0>175 && val_t0<240)) //CASE II: different color but white

{

if (color_t0.equals("red") || color_t0.equals("yellow")) //CASE IIa neighbouring color

{

if (Math.abs(dtheta)>thetaSwitchThreshold && Math.abs(dpsi)>psiSwitchThreshold)

{

val_t1=160; //request high initiation Threshold for switching 160=orange starting value

color_t1="orange";

assignment=true;

} else

{

if (val_t0!=143 && val_t0!=207) //check for penalty value, if not true assign previous color, else white

{

val_t1=val_t0;

color_t1=color_t0;

assignment=true;

} else

{

val_t1=241;

color_t1="white";

assignment=true;

}

}

} else

{

if (color_t0.equals("blue") || color_t0.equals("green") || color_t0.equals("lightgreen") || color_t0.equals("cyan") || color_t0.equals("pink")) //other than neighbouring colors

{

if (chigh2==true) //if high counter: long movement in one direction keep t0 color, assign penalty

{

val_t1=val_t0+11;

color_t1=color_t0;

assignment=true;

} else //if counter low set white

{

val_t1=241;

color_t1="white";

assignment=true;

}

}

}

}

else if (color_t1.equals(color_t0)) //CASE III: same color for t1 and t0

{

if (val_t0<164) val_t1=val_t0+1; //counter limit

else if (val_t0==175) val_t1=160; //important for penalty reset

else val_t1=164;

color_t1="orange";

assignment=true;

}

}

else if (color_t1.equals("red"))

{

if (color_t0.equals("white") || color_t0.equals("black")) //CASE I: color_t0.equals(white

{

if (dpsi>psiEntryThreshold && dtheta<=thetaMainThreshold && dtheta>=-thetaMainThreshold)

{

val_t1=192; //request high initiation Threshold for starting 192=red starting value

color_t1="red";

assignment=true;

} else

{

if (val_t0<10) val_t1=240;

else if (val_t0<244) val_t1=val_t0+1; //Threshold not reached=> assign white using counter

else val_t1=val_t0;

color_t1="white";

assignment=true;

}

}

else if (val_t0<192 || (val_t0>207 && val_t0<240)) //CASE II: different color but white

{

if (color_t0.equals("pink") || color_t0.equals("orange")) //CASE IIa neighbouring color

{

if (Math.abs(dpsi)>psiSwitchThreshold)

{

val_t1=192; //request high initiation Threshold for switching 192=red starting value

color_t1="red";

assignment=true;

} else

{

if (val_t0!=175 && val_t0!=223) //check for penalty value, if not true assign previous color, else white

{

val_t1=val_t0;

color_t1=color_t0;

assignment=true;

} else

{

val_t1=241;

color_t1="white";

assignment=true;

}

}

} else

{

if (color_t0.equals("blue") || color_t0.equals("cyan") || color_t0.equals("green") || color_t0.equals("lightgreen") || color_t0.equals("yellow")) //other than neighbouring colors

{

if (chigh2==true) //if high counter: long movement in one direction keep t0 color, assign penalty

{

val_t1=val_t0+11;

color_t1=color_t0;

assignment=true;

} else //if counter low set white

{

val_t1=241;

color_t1="white";

assignment=true;

}

}

}

}

else if (color_t1.equals(color_t0)) //same color for t1 and t0

{

if (val_t0<196) val_t1=val_t0+1; //counter limit

else if (val_t0==207) val_t1=192; //important for penalty reset

else val_t1=196;

color_t1="red";

assignment=true;

}

}

else if (color_t1.equals("pink"))

{

if (color_t0.equals("white") || color_t0.equals("black")) //CASE I: color_t0.equals(white

{

if (dpsi>psiSwitchThreshold || dtheta<-thetaSwitchThreshold)

{

val_t1=208; //request high initiation Threshold for starting 208=pink starting value

color_t1="pink";

assignment=true;

} else

{

if (val_t0<10) val_t1=240;

else if (val_t0<244) val_t1=val_t0+1; //Threshold not reached=> assign white using counter

else val_t1=val_t0;

color_t1="white";

assignment=true;

}

}

else if (val_t0<208 || (val_t0>223 && val_t0<240)) //CASE II: different color but white

{

if (color_t0.equals("blue") || color_t0.equals("red")) //CASE IIa neighbouring color

{

if (Math.abs(dtheta)>thetaSwitchThreshold && Math.abs(dpsi)>psiSwitchThreshold)

{

val_t1=208; //request high initiation Threshold for switching 208=pink starting value

color_t1="pink";

assignment=true;

} else

{

if (val_t0!=47 && val_t0!=207) //check for penalty value, if not true assign previous color, else white

{

val_t1=val_t0;

color_t1=color_t0;

assignment=true;

} else

{

val_t1=241;

color_t1="white";

assignment=true;

}

}

} else

{

if (color_t0.equals("cyan") || color_t0.equals("green") || color_t0.equals("lightgreen") || color_t0.equals("yellow") || color_t0.equals("orange")) //other than neighbouring colors

{

if (chigh2==true) //if high counter: long movement in one direction keep t0 color, assign penalty

{

val_t1=val_t0+11;

color_t1=color_t0;

assignment=true;

} else //if counter low set white

{

val_t1=241;

color_t1="white";

assignment=true;

}

}

}

}

else if (color_t1.equals(color_t0)) //CASE III: same color for t1 and t0

{

if (val_t0<212) val_t1=val_t0+1; //counter limit

else if (val_t0==223) val_t1=208; //important for penalty reset

else val_t1=212;

color_t1="pink";

assignment=true;

}

}

else if (color_t1.equals("white"))

{

if (color_t0.equals("black"))

{

val_t1=241;

color_t1="white";

assignment=true;

}

if (color_t0.equals("white"))

{

if (val_t0<244) val_t1=val_t0+1;

else val_t1=244;

color_t1="white";

assignment=true;

}

if (color_t0.equals("blue")) //test for direction of t1 in direction of t0 using 20x lower Threshold if true no penalty, counter-1

{

if (dtheta<-0.01 && dpsi<=0.0001 && dpsi>=-0.0001)

{

if (val_t0>=33 && val_t0<=36) val_t1=val_t0-1;

else val_t1=32;

color_t1="blue";

assignment=true;

}

else // if not true assign penalty

{

if (chigh1==true || chigh2==true)

{

val_t1=47;

color_t1="blue";

assignment=true;

}

else if (penalty==true) //check wether penalty value has already been assigned, if true t1 and t0=white

{

val_t0=241;

color_t0="white";

val_t1=241;

color_t1="white";

assignment=true;

}

else if (chigh1==false && chigh2==false) //if counter low assign white

{

val_t1=241;

color_t1="white";

assignment=true;

}

}

}

if (color_t0.equals("cyan"))

{

if (dpsi<-0.0001 && dtheta<-0.01)

{

if (val_t0>=81 && val_t0<=84) val_t1=val_t0-1;

else val_t1=80;

color_t1="cyan";

assignment=true;

}

else // if not true assign penalty

{

if (chigh1==true || chigh2==true)

{

val_t1=95;

color_t1="cyan";

assignment=true;

}

else if (penalty==true) //check wether penalty value has already been assigned, if true t1 and t0=white

{

val_t0=241;

color_t0="white";

val_t1=241;

color_t1="white";

assignment=true;

}

else if (chigh1==false && chigh2==false) //if counter low assign white

{

val_t1=241;

color_t1="white";

assignment=true;

}

}

}

if (color_t0.equals("green"))

{

if (dpsi<-0.0001 && dtheta<=0.01 && dtheta>=-0.01)

{

if (val_t0>=97 && val_t0<=100) val_t1=val_t0-1;

else val_t1=96;

color_t1="green";

assignment=true;

}

else // if not true assign penalty

{

if (chigh1==true || chigh2==true)

{

val_t1=111;

color_t1="green";

assignment=true;

}

else if (penalty==true) //check wether penalty value has already been assigned, if true t1 and t0=white

{

val_t0=241;

color_t0="white";

val_t1=241;

color_t1="white";

assignment=true;

}

else if (chigh1==false && chigh2==false) //if counter low assign white

{

val_t1=241;

color_t1="white";

assignment=true;

}

}

}

if (color_t0.equals("lightgreen"))

{

if (dpsi<-0.0001 && dtheta>0.01)

{

if (val_t0>=113 && val_t0<=116) val_t1=val_t0-1;

else val_t1=112;

color_t1="lightgreen";

assignment=true;

}

else // if not true assign penalty

{

if (chigh1==true || chigh2==true)

{

val_t1=127;

color_t1="lightgreen";

assignment=true;

}

else if (penalty==true) //check wether penalty value has already been assigned, if true t1 and t0=white

{

val_t0=241;

color_t0="white";

val_t1=241;

color_t1="white";

assignment=true;

}

else if (chigh1==false && chigh2==false) //if counter low assign white

{

val_t1=241;

color_t1="white";

assignment=true;

}

}

}

if (color_t0.equals("yellow"))

{

if (dtheta>0.01 && dpsi<=0.0001 && dpsi>=-0.0001)

{

if (val_t0>=129 && val_t0<=132) val_t1=val_t0-1;

else val_t1=128;

color_t1="yellow";

assignment=true;

}

else // if not true assign penalty

{

if (chigh1==true || chigh2==true)

{

val_t1=143;

color_t1="yellow";

assignment=true;

}

else if (penalty==true) //check wether penalty value has already been assigned, if true t1 and t0=white

{

val_t0=241;

color_t0="white";

val_t1=241;

color_t1="white";

assignment=true;

}

else if (chigh1==false && chigh2==false) //if counter low assign white

{

val_t1=241;

color_t1="white";

assignment=true;

}

}

}

if (color_t0.equals("orange"))

{

if (dpsi>0.0001 && dtheta>0.01)

{

if (val_t0>=161 && val_t0<=164) val_t1=val_t0-1;

else val_t1=160;

color_t1="orange";

assignment=true;

}

else // if not true assign penalty

{

if (chigh1==true || chigh2==true)

{

val_t1=175;

color_t1="orange";

assignment=true;

}

else if (penalty==true) //check wether penalty value has already been assigned, if true t1 and t0=white

{

val_t0=241;

color_t0="white";

val_t1=241;

color_t1="white";

assignment=true;

}

else if (chigh1==false && chigh2==false) //if counter low assign white

{

val_t1=241;

color_t1="white";

assignment=true;

}

}

}

if (color_t0.equals("red"))

{

if (dpsi>0.0001 && dtheta<=0.01 && dtheta>=-0.01)

{

if (val_t0>=193 && val_t0<=196) val_t1=val_t0-1;

else val_t1=192;

color_t1="red";

assignment=true;

}

else // if not true assign penalty

{

if (chigh1==true || chigh2==true)

{

val_t1=207;

color_t1="red";

assignment=true;

}

else if (penalty==true) //check wether penalty value has already been assigned, if true t1 and t0=white

{

val_t0=241;

color_t0="white";

val_t1=241;

color_t1="white";

assignment=true;

}

else if (chigh1==false && chigh2==false) //if counter low assign white

{

val_t1=241;

color_t1="white";

assignment=true;

}

}

}

if (color_t0.equals("pink"))

{

if (dpsi>0.0001 && dtheta<-0.01)

{

if (val_t0>=209 && val_t0<=212) val_t1=val_t0-1;

else val_t1=208;

color_t1="pink";

assignment=true;

}

else // if not true assign penalty

{

if (chigh1==true || chigh2==true)

{

val_t1=223;

color_t1="pink";

assignment=true;

}

else if (penalty==true) //check wether penalty value has already been assigned, if true t1 and t0=white

{

val_t0=241;

color_t0="white";

val_t1=241;

color_t1="white";

assignment=true;

}

else if (chigh1==false && chigh2==false) //if counter low assign white

{

val_t1=241;

color_t1="white";

assignment=true;

}

}

}

}

}

else

{

if (dpsi>psiEntryThreshold && dtheta<=thetaEntryThreshold && dtheta>=-thetaEntryThreshold)

{

val_t1=192;

color_t1="red";

}

else if (dpsi>psiEntryThreshold && dtheta>thetaEntryThreshold)

{

val_t1=160;

color_t1="orange";

}

else if (dpsi>psiEntryThreshold && dtheta<-thetaEntryThreshold)

{

val_t1=208;

color_t1="pink";

}

else if (dpsi<-psiEntryThreshold && dtheta<=thetaEntryThreshold && dtheta>=-thetaEntryThreshold)

{

val_t1=96;

color_t1="green";

}

else if (dpsi<-psiEntryThreshold && dtheta>thetaEntryThreshold)

{

val_t1=112;

color_t1="lightgreen";

}

else if (dpsi<-psiEntryThreshold && dtheta<-thetaEntryThreshold)

{

val_t1=80;

color_t1="cyan";

}

else if (dtheta>thetaEntryThreshold && dpsi<=psiEntryThreshold && dpsi>=-psiEntryThreshold)

{

val_t1=128;

color_t1="yellow";

}

else if (dtheta<-thetaEntryThreshold && dpsi<=psiEntryThreshold && dpsi>=-psiEntryThreshold)

{

val_t1=32;

color_t1="blue";

}

else if (dtheta>=-thetaEntryThreshold && dtheta <=thetaEntryThreshold && dpsi>=-psiEntryThreshold && dpsi<=psiEntryThreshold)

{

val_t1=241;

color_t1="white";

}

}

if (color_t1.equals("white")) whitecounter++;

if (color_t1.equals("blue")) bluecounter++;

if (color_t1.equals("cyan")) cyancounter++;

if (color_t1.equals("yellow")) yellowcounter++;

if (color_t1.equals("lightgreen")) lightgreencounter++;

if (color_t1.equals("green")) greencounter++;

if (color_t1.equals("orange")) orangecounter++;

if (color_t1.equals("red")) redcounter++;

if (color_t1.equals("pink")) pinkcounter++;

Framet1_is.setVoxel(xt1,yt1,zt1,val_t1);

/* //Unrolling of embryo

if (flatMap && ventralSide){

x_flat = 500 + (int) ((psi_t1)*circleRadius);

radDiff = circleRadius-theta_t1;

y_flat = 100 + (int) (radDiff);

flatFrameV_is.setVoxel(x_flat, y_flat, zt1, val_t1);

}

if (flatMap && dorsalSide){

if (psi_t1<0) x_flat = 500 + (int) ((-Math.PI-psi_t1)*circleRadius);

else x_flat = 500 + (int) ((Math.PI-psi_t1)*circleRadius);

radDiff = circleRadius-theta_t1;

y_flat = 100 + (int) (radDiff);

flatFrameD_is.setVoxel(x_flat, y_flat, zt1, val_t1);

}

if (flatMap && elongated){

if (psi_t1<0) x_flat = 500 + (int) ((-Math.PI-psi_t1)*circleRadius);

else x_flat = 500 + (int) ((Math.PI-psi_t1)*circleRadius);

radDiff = circleRadius-theta_t1;

y_flat = 100 + (int) (radDiff);

flatFrameEl_is.setVoxel(x_flat, y_flat, zt1, val_t1);

if (x>168) {

x_flat = 500 + (int) ((psi_t1)*circleRadius);

radDiff = circleRadius-theta_t1;

y_flat = 100 + (int) (radDiff);

flatFrameEl_is.setVoxel(x_flat, y_flat, 1850-zt1, val_t1);

}

}*/

//IJ.log("i: "+i+"; x: "+x+"; y: "+y+"; z: "+z+"; dx: "+dx+"; dy: "+dy+"; dz: "+dz+"; xt1: "+xt1+"; yt1: "+yt1+"; zt1: "+zt1+"; val_t1: "+val_t1); //+"; val_t0: "+val_t0);

}

}

}

}

//pixels_t1[i]=(int) val_t1;

//Framet1_is.setSlice(zt1);

IJ.saveAs(Framet1_imp, "Tiff", outputPath+"3D_Embryo/Frame_"+j+".tif");

/*if (flatMap && ventralSide){

IJ.saveAs(flatFrameV_imp, "Tiff", outputPath+"Ventral_split/flatFrameV_"+j+".tif");

}

if (flatMap && dorsalSide){

IJ.saveAs(flatFrameD_imp, "Tiff", outputPath+"Dorsal_split/flatFrameD_"+j+".tif");

}

if (flatMap && elongated){

IJ.saveAs(flatFrameEl_imp, "Tiff", outputPath+"Elongated/flatFrameEl_"+j+".tif");

}*/

Frame1=false;

IJ.log("Red: "+redcounter+" Green: "+greencounter+" Blue: "+bluecounter+" Yellow: "+yellowcounter+" Pink: "+pinkcounter+" Orange: "+orangecounter+" Cyan: "+cyancounter+" Lightgreen: "+lightgreencounter);

}

}

}
